# Identifying women giving birth preterm and care at the time of birth: a prospective audit of births at six hospitals in India, Kenya, Pakistan and Uganda

**DOI:** 10.1186/s12884-020-03126-0

**Published:** 2020-07-31

**Authors:** Eleanor J. Mitchell, Santosh Benjamin, Sam Ononge, James Ditai, Zahida Qureshi, Shabeen Naz Masood, Diane Whitham, Peter J. Godolphin, Lelia Duley, on behalf of the “iCord Collaborative Group”, Diane Whitham, Eleanor J. Mitchell, Peter J. Godolphin, Lelia Duley, Santosh Benjamin, Jiji Mathews, Manish Kumar, K. Anil Kuruvilla, M. Nirmala, Nayana John, Sam Ononge, Mariam Nabwire, Rose Mary Byenkya, Emilly Nakinja, Grace Engeu Ariokot, Sarah Namuddu, James Ditai, Stephen Waiswa, Macreen Mudoola, Auma Proscovia, Julian Abesco, Susan Magoba, Zahida Qureshi, Alfred Osoti, Rachel Musoke, Raheli Mukhwana, Shabeen Naz Masood, Zehra Naqvi, Haleema A. Hashmi, Samina Shamim, Syed Kashif Abbas Zaidi, Yasir Masood

**Affiliations:** 1 Nottingham Clinical Trials Unit, University Of Nottingham, Nottingham, UK; 2 Christian Medical College, Vellore, India; 3 Makerere University College of Health Science, Kampala, Uganda; 4 Sanyu Africa Research Institute, Mbale, Uganda; 5 University of Nairobi, Nairobi, Kenya; 6 Isra Medical University, Karachi-Campus, Karachi, Pakistan

**Keywords:** Low and middle income countries, Cord clamping, Prospective audit, Neonatal care, Preterm birth

## Abstract

**Background:**

Globally, 15 million infants are born preterm each year, and 1 million die due to complications of prematurity. Over 60% of preterm births occur in Sub-Saharan Africa and south Asia. Care at birth for premature infants may be critical for survival and long term outcome. We conducted a prospective audit to assess whether women giving birth preterm could be identified, and to describe cord clamping and neonatal care at hospitals in Africa and south Asia.

**Methods:**

This prospective audit of livebirths was conducted at six hospitals in Uganda, Kenya, India and Pakistan. Births were considered preterm if between 28^+ 0^ and 33^+ 6^ weeks gestation and/or the birthweight was 1.00 to 1.99 kg. A pre-specified audit plan was agreed with each hospital. Livebirths before 28 weeks gestation with birthweight less than 1.0 kg were excluded. Data were collected on estimated and actual gestation and birthweight, cord clamping, and neonatal care.

**Results:**

Of 4149 women who gave birth during the audit, data were available for 3687 (90%). As 107 were multiple births, 3781 livebirths were included, of which 257 (7%) were preterm. Antenatal assessment correctly identified 148 infants as ‘preterm’ and 3429 as ‘term’, giving a positive predictive value of 72% and negative predictive value of 97%. For term births, cord clamping was usually later at the two Ugandan hospitals, median time to clamping 50 and 76 s, compared with 23 at Kenyatta (Kenya), 7 at CMC (India) and 12 at FBH/LNH (Pakistan). At the latter two, timing was similar between term and preterm births, and between vaginal and Caesarean births. For all the hospitals, the cord was clamped quickly at Caesarean births, with Mbale (Uganda) having the highest median time to clamping (15 s ‘term’, 19 ‘preterm’). For preterm infants temperature on admission to the neonatal unit was below 35.5 °C for 50%, and 59 (23%) died before hospital discharge.

**Conclusions:**

Antenatal identification of preterm birth was good. Timing of cord clamping varied between hospitals, although at each there was no difference between ‘term’ and ‘preterm’ births. For premature infants hypothermia was common, and mortality before hospital discharge was high.

## Background

Being born preterm (before 37 weeks gestation) has major impact on survival and quality of life for the child, on psychosocial and emotional stress on the family, and on costs for health services and society [1–3]. Globally, around 15 million infants are born preterm each year, of whom more than 1 million die due to complications of prematurity [4, 5]. Over 80% of preterm births occur in sub-Saharan Africa and south Asia [5]. Twelve percent of newborns in low income settings are preterm compared to 9% in high income countries [5]. Amongst children born very preterm (before 32 weeks) who survive, morbidity is particularly high compared to those born at term [3]; in Europe, for example, around 5% develop cerebral palsy, and those without severe disability have a two-fold or greater increased risk for developmental, cognitive, and behavioural difficulties [1, 2]. These impairments may persist into adolescence and early adulthood [6, 7]. Reducing the morbidity and mortality associated with preterm birth is a priority [4, 8].

Care at birth, particularly for infants born preterm or sick, may be critical for survival and long term outcome. Optimal timing for umbilical cord clamping for both term and preterm births is controversial [9]. Traditionally, immediate cord clamping was widely implemented as part of active management of the third stage of labour [10]. For healthy term births, the evidence now supports a more liberal approach to delaying cord clamping [11], and WHO recommends clamping between 1 and 3 min [12]. Delayed cord clamping allows longer for the cardiorespiratory changes that occur at birth as the neonatal circulation is established, hence it may potentially be of greater benefit for preterm infants with immature lungs and myocardium [13]. For preterm births the evidence on when is best to clamp the cord is unclear, however, and trials have largely been conducted at births before 32 to 34 weeks in settings with access to neonatal intensive care [14, 15]. Yet delayed cord clamping may have additional advantages in low resource settings where neonatal care is limited or unavailable [16].

Our objectives were to describe care at the time of preterm birth in low and middle income hospital settings, and to assess whether a randomised trial of alternative policies for timing of cord clamping at preterm birth might be feasible. Assessment of gestation in many maternity units in low and middle income settings often relies primarily on menstrual history and clinical examination. Therefore, we conducted a prospective audit to assess whether women giving birth preterm could be identified, and to describe timing of cord clamping and neonatal care.

## Methods

This prospective audit was conducted between May and September 2015 at six hospitals in four low and middle income countries: Mulago National Referral Hospital, Kampala and Mbale Regional Referral Hospital in Uganda; Kenyatta National Hospital, Nairobi, Kenya; Christian Medical College (CMC), Vellore, India; and Fatima Bai Hospital and Liaquat National Hospital (FBH/LNH), Karachi, Pakistan. These hospitals were selected based on their interest in conducting a randomized trial evaluating alternative strategies for the timing of cord clamping at preterm birth. Hospitals varied in terms of available resources and funding/management structures, though this was not considered for selection purposes. At one hospital (Mbale), a neonatal unit was in the process of being established and there was no capacity for mechanical ventilation. The other five hospitals had some form of neonatal unit with some capacity to provide mechanical ventilation. All the hospitals had busy maternity units, with high levels of preterm birth and perinatal mortality. Ethics approval for each hospital was obtained according to local requirements. The study was observational, with all aspects of care being according to local practice.

For the purpose of this audit, we focused on preterm births associated with high mortality and morbidity. Therefore, we considered births as preterm if they were between 28^+ 0^ weeks and 33^+ 6^ weeks gestation or the birthweight was 1.00 kg to 1.99 kg; and as ‘term’ if they were 34^+ 0^ weeks or above and birthweight was 2 kg or more. We used 34 weeks as the upper gestational age cut off as above this mortality is low and the infants usually do not require admission to a neonatal unit. As menstrual history is often unknown, and can be unreliable for assessing gestation, and a dating ultrasound scan is not usually available, we included birthweight as a surrogate for gestation, a common practice at the participating hospitals and more widley in low and middle income countries. For all infants admitted to a neonatal unit we requested a gestational age assessment using either the Dubowitz [17] or New Ballard [18], whichever was in local practice.

The aim was to collect data for at least 30 preterm births at each hospital, which we estimated would require surveillance of 500–700 births. Continuous surveillance was not realistic in these settings, so we agreed a pre-specified plan for when the audit would be conducted within each hospital. To ensure a representative sample, this included day and night, weekdays and weekends, and was across different staff shifts. During each pre-specified audit period all livebirths on the delivery unit were eligible to be included. Births before the woman arrived on the delivery unit, stillbirths, and livebirths before 28 weeks gestation with birthweight less than 1 kg were excluded. Before the audit began at each hospital, the local staff who would be conducting the study attended 3 days of training in study procedures. As time of cord clamping and some other delivery room care is not usually recorded in the clinical notes, this included training in observing the birth, timing events with a stop watch, and recording the information. Staff were also trained in how to extract relevant information from the clinical notes onto our data collection forms, and to enter the data onto the database.

During each pre-specified audit period, we collected data on: estimated gestation and how this was estimated (last menstrual period (LMP), clinical assessment, and/or dating ultrasound scan as per local practice); estimated birthweight and how this was estimated (clinical assessment or ultrasound scan); assessment of gestation after birth, and how this was assessed (LMP, clinical assessment, dating ultrasound scan, a Dubowitz [17] or New Ballard [18] assessment); birthweight; and timing of cord clamping. For births that met our criteria for preterm we also collected data on: neonatal care at birth; admission to the neonatal unit or paediatric ward, and duration of stay; serious neonatal morbidity, and outcome at discharge from hospital. Delivery unit records were checked for eligible births missed during the audit period, and for these data were collected on gestation at birth, birthweight, and mode of birth.

Data were anonymous, with each woman and her infant(s) identified by a unique study ID. At each hospital, data were entered into a secure online database developed and maintained by the Nottingham Clinical Trials Unit.

### Analysis

For each hospital we described: births missed during the audit period; estimated gestation at birth and birthweight, (and how these were estimated, i.e. LMP, clinical assessment, dating ultrasound scan); timing of cord clamping for ‘preterm’ and ‘term’ births; the number of births identified as preterm before birth; clinical staff present at these preterm births; neonatal care provided at birth and after birth; and serious neonatal morbidity. Mortality was described both overall and per site; analysis by timing of cord clamping and mortality was not performed due to the observational study design and risk of bias. We classified neonatal units as: Level 1 if they had no access to ventilation support, Level 2 if there was limited access to ventilation support, and Level 3 if they provided neonatal intensive care including the capacity to provide prolonged mechanical ventilation [19, 20]. Level 3 units were available at the hospitals in India, Kenya and Pakistan, but not in Uganda.

For assessment of gestation before birth, we calculated the positive predictive value (PPV) and negative predictive value (NPV) of being preterm. If data were missing for either gestation or birthweight, then the single measure (gestation or birthweight) was used. If data for both gestation and birthweight was missing, the infant was excluded from the PPV/NPV calculation. We conducted three sensitivity analyses: using only infants with complete data for gestation and birthweight; categorising infants with missing birthweight solely on gestation; and assuming that all infants with missing data were born at term.

Variables were described as median (interquartile range) or mean (standard deviation) when continuous, and N (%) when categorical. All analyses were performed in Stata version 14.2 or later.

## Results

**Fig. 1:**
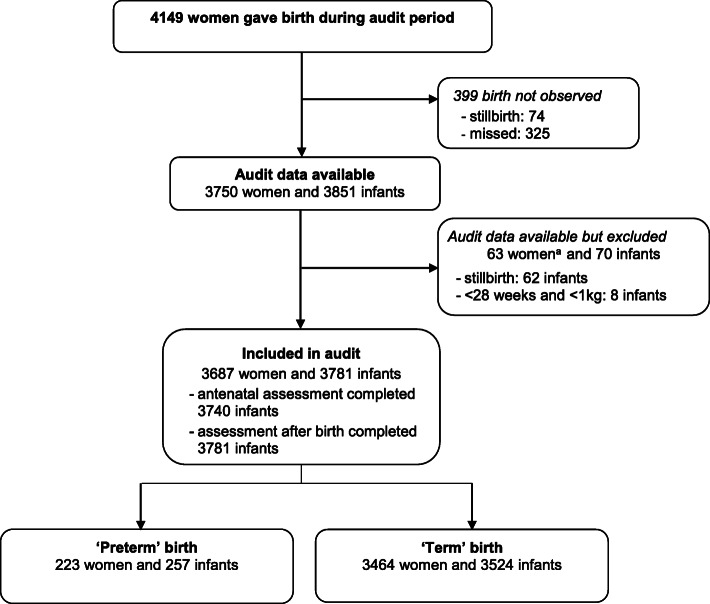
Flow chart for audit of births. ^a^5 women had a multiple birth with at least 1 infant not eligible (due to stillbirth or birthweight < 1 kg) and at least one infant eligible (due to livebirth or birthweight ≥1 kg). Therefore, these five women are not excluded

Overall, 4149 women gave birth during the audit period; 399 of these births were not observed; for 74 women this was because she was known to be having a stillbirth (Fig. 1).

Of the 325 women with a livebirth for whom no audit data were available, 7 (2%) had a preterm birth, 241 (74%) a spontaneous vaginal birth, 80 (25%) caesarean section and 4 (1%) a vaginal breech or instrumental birth. Audit data were available for 3750 (90%) women, of whom 63 were excluded (Fig. 1). Of the 3687 women included in the audit, 3580 had a singleton pregnancy and 107 a multiple pregnancy. For five women who had a multiple pregnancy, at least one infant was eligible and included in the audit and at least one infant was not eligible (based on birthweight or stillbirth).In the audit, 3687 women gave birth to 3781 live newborns, of whom 257 were ‘preterm’ and 3524 were ‘term’ (Fig. 1).

Before birth an estimate of gestation was available for 99% of the women; for three quarters this was based on the LMP and/or clinical assessment (Table 1). For one in five women the estimate of gestational age was based on a dating ultrasound scan, but these women were mostly in CMC (India) and FBH/LNH (Pakistan). Of the 227 births considered preterm at the time of birth, 181 (80%) had a neonatal assessment for gestational age. Overall birthweight was estimated before birth for a quarter of the infants, but this ranged from 3% of births in Mbale (Uganda) to 90% in CMC (India). For almost three quarters of the infants with an estimated birthweight (652/900, 72%), this estimate was based on clinical assessment alone (Table 2).

**Table 1:** For all livebirths included in the audit, estimated gestation before birth and assessment of gestation after birth

	CMC (*n* = 692)		Mulago (*n* = 958)		Mbale (*n* = 772)		Kenyatta (*n* = 992)		FBH/LNH (*n* = 273)		Total (*n* = 3687)	
***Estimated before birth***	691	(99%)	957	(99%)	770	(99%)	954	(96%)	272	(99%)	**3644**	**(99%)**
*Method*												
LMP and/or clinical assessment	191	(28%)	855	(89%)	742	(96%)	921	(93%)	169	(62%)	**2878**	**(78%)**
Dating ultrasound scan alone	211	(30%)	55	(6%)	21	(3%)	31	(3%)	37	(14%)	**355**	**(10%)**
Dating ultrasound scan + other	289	(42%)	47	(5%)	7	(1%)	2	(< 1%)	66	(24%)	**411**	**(11%)**
*Gestation (weeks)*												
< 28	–	–	11	(1%)	4	(1%)	10	(1%)	3	(1%)	**28**	**(1%)**
28–33^+ 6^	14	(2%)	52	(5%)	26	(3%)	36	(4%)	17	(6%)	**145**	**(4%)**
≥ 34	677	(98%)	894	(93%)	740	(96%)	908	(92%)	252	(92%)	**3471**	**(94%)**
***Assessed after birth***												
*Gestation (weeks)*												
< 28	–	–	3	(< 1%)	–	–	3	(< 1%)	2	(1%)	**8**	**(< 1%)**
28–33^+ 6^	15	(2%)	67	(7%)	10	(1%)	29	(3%)	15	(5%)	**136**	**(4%)**
≥ 34	677	(98%)	888	(93%)	762	(99%)	960	(97%)	256	(94%)	**3543**	**(96%)**
***Neonatal assessment done***^***a***^	-^b^		81		32		45		23		**181**	

All data are N (%)

*LMP* Last menstrual period

^a^Neonatal Assessments were only done for births thought to be preterm, CMC (*n* = 39), Mulago (*n* = 82), Mbale (*n* = 32), Kenyatta (*n* = 49), FBH/LNH (*n* = 25), Total (*n* = 227)

^b^Not reported as only done for the infants for whom the neonatologist thought there was a discrepancy between before and after birth assessments

**Table 2:** For all livebirths in the audit, birthweight estimated before birth and actual birthweight

	CMC (*n* = 703)		Mulago (*n* = 993)		Mbale (*n* = 787)		Kenyatta (*n* = 1018)		FBH/LNH (*n* = 280)		Total (*n* = 3781)	
***Birthweight estimate***	632	(90%)	89	(9%)	22	(3%)	51	(5%)	106	(38%)	**900**	**(24%)**
*Method*												
Clinical assessment	619	–	5	–	–	–	1	–	27	–	**652**	**–**
Ultrasound scan	13	–	84	–	22	–	50	–	79	–	**248**	**–**
*Estimate (kg)*												
< 1.00	–	–	–	–	1	–	–	–	2	–	**3**	**–**
1.00–1.99	36	–	15	–	–	–	12	–	15	–	**78**	**–**
2.00–2.99	354	–	17	–	8	–	16	–	56	–	**451**	**–**
> 3.00	242	–	57	–	13	–	23	–	33	–	**368**	**–**
***Actual birthweight****(kg)*												
< 1.00	–	–	6	(1%)	1	(< 1%)	1	(< 1%)	1	(< 1%)	**9**	**(< 1%)**
1.00–1.99	45	(6%)	68	(7%)	35	(4%)	52	(5%)	16	(6%)	**216**	**(6%)**
2.00–2.99	327	(47%)	303	(31%)	318	(40%)	323	(32%)	108	(39%)	**1379**	**(36%)**
> 3.00	331	(47%)	616	(62%)	433	(55%)	642	(63%)	155	(55%)	**2177**	**(57%)**

All data are N (%)

**Fig. 2:**
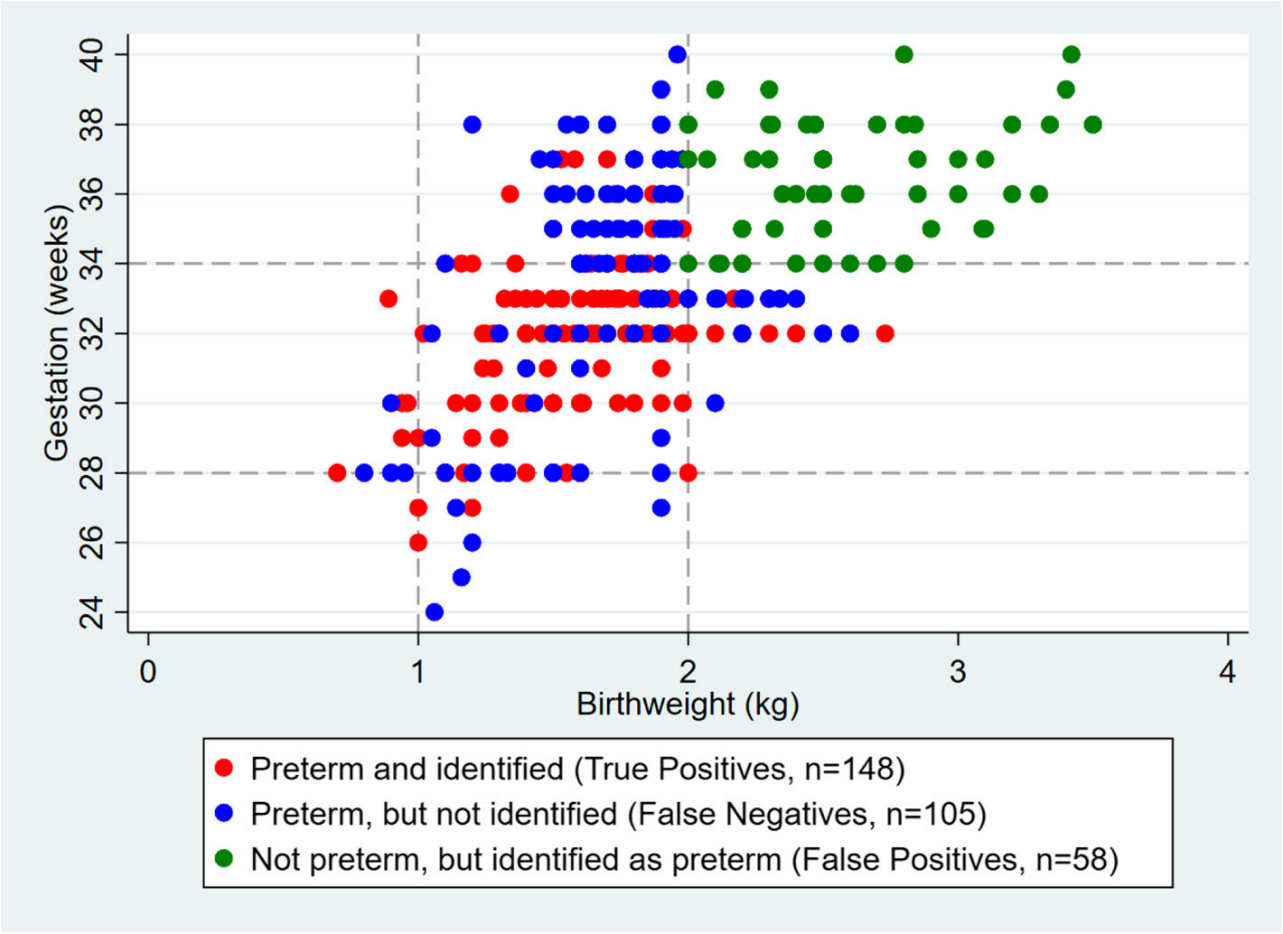
Identification of infants born preterm using antenatal assessments and confirmation using postnatal assessment

Comparing antenatal assessment with assessment of gestation and birthweight after birth for the 3740 infants with these data available, 148 (4%) were correctly identified as preterm and 3429 (92%) were correctly recognised as term (Fig. 2 and *Supplementary material*, Table S1). This corresponds to a PPV of 72% and NPV of 97% for assessment before birth. The three sensitivity analyses produced identical PPV’s, with NPV only differing for one sensitivity analysis to 96%.

Just over one third (37%) of the total births were by Caesarean section (Table 3), ranging from 22% at Mbale (Uganda) to 61% at FBH/LNH (Pakistan). For term births, cord clamping was usually later at the two hospitals in Uganda, with median time to clamping 50 and 76 s, compared with 7 s at CMC (India) and 12 s at FBH/LNH s (Pakistan). At these two latter hospitals, timing of cord clamping was similar between ‘term’ and ‘preterm births’, and between vaginal and Caesarean births (Table 3). For all the hospitals, the cord was clamped quickly at Caesarean births, with Mbale (Uganda) having the highest median time to clamping of 15 s for term birth and 19 s for preterm births (Table 4, and *Supplementary material*, Figure S1).

**Table 3:** Mode of birth and timing of cord clamping for ‘term’ and ‘preterm’ births

	CMC (*n* = 703)		Mulago (*n* = 993)		Mbale (*n* = 787)		Kenyatta (*n* = 1018)		FBH/LNH (*n* = 280)		Total (*n* = 3781)	
***‘Term’***	657		900		750		961		256		**3524**	
Caesarean section	202	(31%)	312	(35%)	165	(22%)	423	(44%)	155	(61%)	**1257**	**(36%)**
Vaginal	455	(69%)	588	(65%)	585	(78%)	538	(56%)	101	(39%)	**2267**	**(64%)**
***Time of cord clamping (seconds)***												
Median {25, 75}	7	{5, 11}	49.5	{11, 87.5}	75.5	{37, 109}	23	{9, 49}	12	{8.5, 17}	**22**	**{8, 69}**
*Caesarean section:* Median {25, 75}	5	{4, 6}	8	{5, 12}	15	{11, 24}	9	{6, 14}	10	{8, 15}	**8**	**{5, 14}**
*Vaginal:* Median {25, 75}	8	{6, 12}	76.5	{50, 109.5}	87	{62, 120}	43.5	{25, 62}	15	{11, 20}	**52**	**{17, 88}**
***‘Preterm’***	46		93		37		57		24		**257**	
Caesarean section	37	(80%)	35	(38%)	7	(19%)	32	(56%)	16	(67%)	**127**	**(49%)**
Vaginal	9	(20%)	58	(62%)	30	(81%)	25	(44%)	8	(33%)	**130**	**(51%)**
***Time of cord clamping (seconds)***												
Median {25, 75}	5	{4, 7}	28	{7, 59}	75	{47, 109}	12	{8, 42}	10.5	{7, 15.5}	**16**	**{6, 52}**
*Caesarean section:* Median {25, 75}	4	{4, 6}	6	{5, 9}	19	{13, 30}	10	{6, 11}	9.5	{6.5, 14}	**7**	**{4, 11}**
*Vaginal:* Median {25, 75}	7	{6, 9}	50	{29, 70}	83	{61, 129}	46	{27, 69}	14	{8, 36.5}	**51**	**{27, 79}**

All data are N (%) unless otherwise stated

**Table 4:** For ‘preterm’ infants, admission to neonatal unit, care in the neonatal unit and outcome at discharge

	CMC (*n* = 46)^a^		Mulago (*n* = 93)		Mbale (*n* = 37)		Kenyatta (*n* = 57)		FBH/LNH (*n* = 24)^a^		Total (*n* = 257)^**a**^	
**Level of neonatal care after birth**												
Postnatal ward	1	(2%)	2	(2%)	7	(19%)	1	(2%)	2	(8%)	**13**	**(5%)**
Neonatal unit, Level 1	–	–	–	–	30	(81%)	–	–	7	(29%)	**37**	**(14%)**
Neonatal unit, Level 2	30	(65%)	91	(98%)	–	–	55	(96%)	–	–	**176**	**(68%)**
Neonatal unit, Level 3	14	(30%)	-^b^	–	-^b^	–	1	(2%)	14	(58%)	**29**	**(11%)**
**Temperature on admission (°C)**												
Mean [SD]	36.1	[0.7]	34.7	[0.8]	35.6	[0.9]	35.5	[0.8]	37	[0.2]	**35.5**	**[1.0]**
< 34.5	–	–	27	(29%)	6	(16%)	3	(5%)	–	–	**36**	**(14%)**
34.5–35.4	8	(17%)	52	(56%)	7	(19%)	26	(46%)	–	–	**93**	**(36%)**
35.5–36.4	21	(46%)	14	(15%)	18	(49%)	19	(33%)	1	(4%)	**73**	**(28%)**
36.5–37.4	14	(30%)	–	–	5	(14%)	6	(11%)	20	(83%)	**45**	**(18%)**
≥ 37.5	1	(2%)	–	–	–	–	2	(4%)	–	–	**3**	**(1%)**
Not known	2	(4%)	–	–	1	(3%)	1	(2%)	3	(13%)	**7**	**(3%)**
**Respiratory support**												
Any respiratory support	5	–	89	–	8	–	52	–	5	–	**159**	**–**
*Type of support and duration (days)*												
Oxygen	1	–	71	–	4	–	46	–	–	–	**122**	**–**
Median {25, 75}	1	{1, 1}	2	{1, 3}	3	{3, 3.5}	4	{3, 6}	–	–	**3**	**{2, 4}**
CPAP	4	–	46	–	1	–	2	–	–	–	**53**	**–**
Median {25, 75}	4	{2.5, 5}	2	{1.5, 4}	2	{2,2}	3	{3, 3}	–	–	**2**	**{2, 4}**
*Ventilation*	1	–	1	–	3	–	5	–	5	–	**15**	**–**
Median {25, 75}	2	{2, 2}	1	{1, 1}	4	{3, 7}	3	{3, 5}	1	{1, 3}	**3**	**{1, 5}**
**Cranial ultrasound**	13	–	2	–	–	–	–	–	–	–	**15**	**–**
IVH	5	–	1	–	–	–	–	–	–	–	**6**	**–**
Duration of stay in neonatal care (days)^*^												
Median {25, 75}	8.5	{5, 25.5}	4	{2, 7}	1	{1, 4}	5	{2.5, 22}	3	{1, 6}	**4.5**	**{2, 8}**
Min, max	1, 66		< 1, 47		< 1, 19		< 1, 102		< 1, 16		**< 1, 102**	
< 1	–	–	4	(4%)	5	(14%)	6	(11%)	3	(13%)	**18**	**(7%)**
1–2	3	(7%)	20	(22%)	16	(43%)	8	(14%)	7	(29%)	**54**	**(21%)**
3–6	12	(26%)	42	(45%)	9	(24%)	17	(30%)	7	(29%)	**87**	**(34%)**
7–14	12	(26%)	24	(26%)	5	(14%)	5	(9%)	3	(13%)	**49**	**(19%)**
≥ 15	17	(37%)	3	(3%)	1	(3%)	20	(35%)	1	(4%)	**42**	**(16%)**
**After leaving neonatal care, discharged**												
Home	36	(78%)	85	(91%)	29	(78%)	54	(95%)	12	(50%)	**216**	**(84%)**
To another ward in the hospital	8	(17%)	8	(9%)	7	(19%)	2	(4%)	9	(38%)	**34**	**(13%)**
Not known	2	(4%)	–	–	1	(3%)	1	(2%)	3	(13%)	**7**	**(3%)**
**Outcome at discharge from hospital**												
Early neonatal death (0–6 days)	1	–	12	–	3	–	27	–	4	–	**47**	**–**
Late neonatal death (7–27 days)	–	–	4	–	1	–	5	–	1	–	**11**	**–**
Post neonatal death (> 27 days)	–	–	–	–	–	–	1	–	–	–	**1**	**–**

All data are N (%) unless otherwise stated

^a^One women at CMC and one women at FBH/LNH were transferred to different hospitals, resulting in missing outcome data

^b^Level 3 neonatal care is not provided in Uganda

For the 130 preterm infants born vaginally, one member of staff was present at the birth for 90, and for all but three births this was a midwife (data not shown). A paediatrician or neonatologist was present for just over a quarter of all preterm births (67, 26%), and for the majority this was in CMC (India) (45, 98%) or FBH/LNH (Pakistan) (19, 79%). Of these preterm infants, 230 (89%) had some neonatal care in the delivery room, the most common being wrapped in plastic or Embrace (www.embraceinnovations.com) (52%), airway suction (54%) and oxygen (54%) (data not shown).

Thirteen (5%) of the preterm infants were taken to the postnatal ward and not admitted to a neonatal unit (Table 4). For the preterm infants, temperature on admission to the neonatal unit or postnatal ward was below 35.5 °C for 50%, with FBH/LNH (Pakistan) the only hospital with no temperatures at this level. Infants appeared coldest in Mulago, where 27 (29%) had a temperature below 34.5 °C. Use of respiratory support varied considerably between hospitals, ranging from 11% of preterm infants in CMC (India) to 96% of those in Mulago (Uganda) (Table 4). Length of stay in the neonatal unit and hospital also varied between hospitals, with median stay of 1 day in Mbale (Uganda) and 8.5 days in CMC (India) (Table 4). Almost a quarter of the preterm infants died before hospital discharge (59, 23%), with mortality ranging from 2% in CMC (India) to 58% at Kenyatta (Kenya).

## Discussion

In order to conduct research to improve the quality of delivery room care for preterm infants it is important to be able to correctly identify those likely to be born preterm. In this prospective audit conducted in delivery units at a range of hospitals in low and middle income countries, we found that using information from antenatal assessments of estimated gestational age and birthweight resulted in reasonable identification of those giving birth preterm (based on our definition using gestational age and/or birthweight). Overall, the PPV was 72% and NPV 97%. Timing of cord clamping varied between hospitals, although within each hospital there was no real difference between timing of cord clamping for ‘term’ and ‘preterm’ births. At all hospitals, for caesarean births cord clamping was largely immediate (within 30 s). For vaginal births, cord clamping was usually immediate at CMC (India) and FBH/LNH (Pakistan), and usually deferred at Mulago and Mbale in Uganda and Kenyatta (Kenya). With the exception of FBH/LNH (Pakistan), hypothermia following preterm birth was common, with half the preterm infants having a temperature below 35.5 °C, and 14% below 34.5 °C on admission to the neonatal unit. Although the numbers are relatively small, our study also confirms the high mortality associated with preterm birth in these settings.

Recently, the evidence base for alternative policies for timing of cord clamping at very preterm birth has increased, including publication of a large international trial co-ordinated from Australia [21] and our own UK trial [22]. A recent systematic review (18 trials, 2834 infants) concluded that ‘delayed clamping reduced hospital mortality’ [15]. There was no clear impact on neonatal or maternal morbidity, but the review was ‘substantially underpowered’ for these outcomes. The review authors also highlighted the need for follow up of the infants in childhood. However, relatively few births included in this review occurred in low and middle income settings; as six studies conducted in Iran [23], India [24, 25], South Africa [26, 27] and Thailand [28] recruited a total of 507 infants, and the international trial included 71 infants recruited in Pakistan [21]. Also, it is likely that these studies were conducted in hospitals with access to a neonatal unit, and with neonatal care available in the delivery room. The comparative effects, both benefits and harms, associated with alternative policies for timing of cord clamping at preterm birth, particularly if very preterm, may be different in settings with limited access to neonatal care. Further evaluation in settings with limited or no access to neonatal care is therefore merited.

This study has shown that women giving birth preterm can be identified in these settings, and so conducting a randomised trial evaluating delivery room interventions for these births would potentially be feasible. Improving assessment of gestational age before birth would increase the proportion of potentially eligible women correctly identified. A future trial design would need to consider the implications of the PPV and NPV reported here and anticipate the inclusion of some births that were thought to be preterm and so randomised, but after birth are assessed as being term.

Despite conducting the audit in places with a warm climate, a surprisingly high proportion of the preterm infants were cold by the time they arrived at the neonatal unit. Keeping newborns warm, particularly those born preterm, is a key element in newborn life support [29], as this reduces morbidity [30, 31]. Common strategies on maternity units in high income settings, such as drying and the use of plastic wraps or bags and external heat sources, are largely unavailable on delivery units in low income settings. Evaluation of low cost interventions to reduce hypothermia, including staff training and availability of appropriate towels or cloths for drying and wrapping the infant following preterm birth in such settings is clearly a priority.

Cranial ultrasound scans were performed on a small number of infants at two of the hospitals in our study. This is not surprising as such scans are often unavailable in low and middle income settings [32].

Strengths of this study are that data were collected prospectively, with births observed to collect information not routinely available in the clinical notes; that we ensured a representative sample of all births was included; that data were available for 90% of births; and that we included a range of hospitals from four countries in low and middle income settings from four countries in Africa and south Asia. Limitations are that although the study was observational, conducting it may have changed care, as at some sites delivery unit midwives were trained to conduct the audit. Therefore they might also have been doing clinical shifts providing care at births included in the audit. Also, just the presence of an observer may have influenced care. As is common for audit data collected from clinical records, we were not able to assess the quality of these data. As our definition of preterm included birthweight, infants who were small for gestational age may potentially have been misclassified, although if a neonatal assessment was available this misclassification could be avoided. Finally, the hospitals contributing data may not be typical of wider practice within each country. Nevertheless, we consider that we included a representative range of units within low and middle income settings where conducting research at the time of preterm birth is feasible.

## Conclusions

Identification of women having a preterm birth, using our definition based on gestational age and/or birthweight, was good, with a PPV of 72% and a NPV of 97% based on antenatal assessment. Therefore conducting research, including randomised trials, to improve the quality of delivery room care for very preterm births would be feasible in hospitals in low and middle income countries. Timing of cord clamping varied between hospitals, although within each hospital there was no difference between ‘term’ and ‘preterm’ births. For caesarean births cord clamping was largely immediate. For vaginal births, timing varied. This variation in practice suggests a randomised trial comparing alternative policies for cord clamping may be feasible. The high levels of hypothermia amongst infants born preterm also suggests that implementing strategies to ensure these infants are kept warm following birth should be a priority. Given the high level of variation in practice in the delivery room and neonatal units, conducting quality improvement initiatives to promote high quality newborn care is also important.

## Supplementary information

**Additional file 1: Figure S1.** Timing of cord clamping for term and preterm births by mode of delivery.

**Additional file 2: Table S1.** Identification of preterm and term infants in the audit.

## Data Availability

The datasets used and analysed during the current study are available from the corresponding author on reasonable request.

## Abbreviations

IVH: Interventricular haemorrhage
CMC: Christian Medical College
FBH/LNH: Fatima Bai Hospital and Liaquat National Hospital
LMP: Last menstrual period
PPV: Positive predictive value
NPV: Negative predictive value
