# Supplementary material for: Identifying women giving birth preterm and care at the time of birth: a prospective audit of births at six hospitals in India, Kenya, Pakistan and Uganda
Source: BMC Pregnancy Childbirth. 2020 Jul 31;20:439. doi: 10.1186/s12884-020-03126-0 (PMC7393815; doi:10.1186/s12884-020-03126-0)

**Supplementary Material:**

**Figure S1:** Timing of cord clamping for term and preterm infants by mode of delivery and hospital


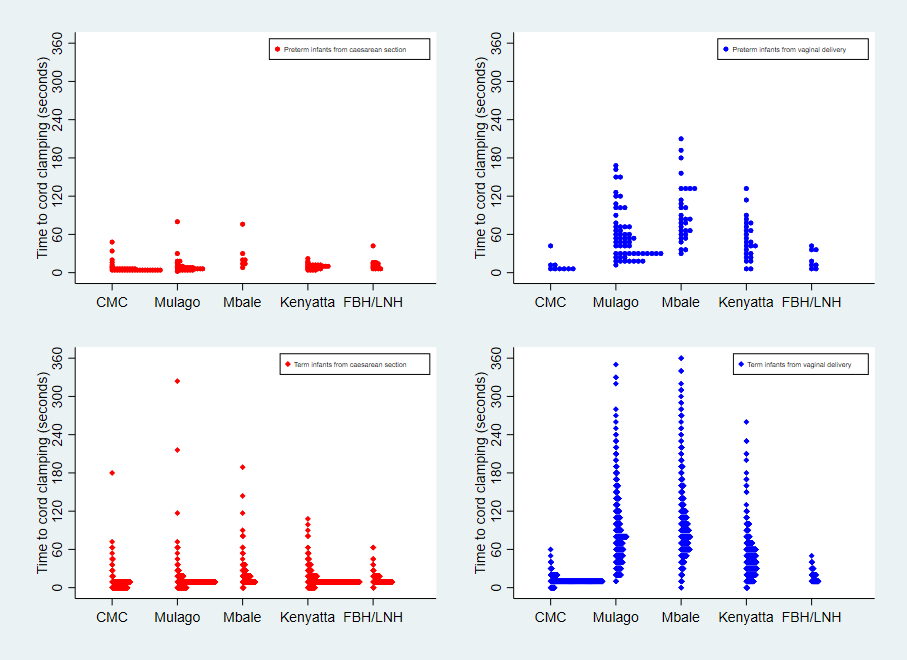

Supplement: Supplementary file 1 — Additional file 1: Figure S1. Timing of cord clamping for term and preterm births by mode of delivery. [file 12884_2020_3126_MOESM1_ESM.docx]
