# Supplementary material for: Identifying women giving birth preterm and care at the time of birth: a prospective audit of births at six hospitals in India, Kenya, Pakistan and Uganda
Source: BMC Pregnancy Childbirth. 2020 Jul 31;20:439. doi: 10.1186/s12884-020-03126-0 (PMC7393815; doi:10.1186/s12884-020-03126-0)
Supplement: Supplementary file 2 — Additional file 2: Table S1. Identification of preterm and term infants in the audit. [file 12884_2020_3126_MOESM2_ESM.docx]

**Table S1:** Identification of preterm and term babies in the audit

*Assumption - babies categorisation is based on gestation if birthweight is unknown – those that have unknown gestation are removed (n=41)*

|  | **Postnatal** | |  |
| --- | --- | --- | --- |
| **Antenatal** | Preterm | Not Preterm | Total |
| Preterm | 148 (TP) | 58 (FP) | 206 |
| Not Preterm | 105 (FN) | 3429 (TN) | 3534 |
| Total | 253 | 3487 | 3740 |
